# Intra- and inter-host origin, evolution dynamics and spatial-temporal transmission characteristics of circoviruses

**DOI:** 10.3389/fimmu.2024.1332444

**Published:** 2024-08-02

**Authors:** Yongqiu Cui, Siting Li, Weiying Xu, Jiali Xie, Dedong Wang, Lei Hou, Jianwei Zhou, Xufei Feng, Jue Liu

**Affiliations:** ^1^ College of Veterinary Medicine, Yangzhou University, Yangzhou, China; ^2^ Jiangsu Co-Innovation Center for Prevention and Control of Important Animal Infectious Diseases and Zoonoses, Yangzhou University, Yangzhou, China

**Keywords:** circovirus, evolutionary origin, phylodynamics, transmission, genetic recombination

## Abstract

**Introduction:**

Since their identification in 1974, circoviruses have caused clinicopathological diseases in various animal species, including humans. However, their origin, transmission, and genetic evolution remain poorly understood.

**Methods:**

In this study, the genome sequences of circovirus were obtained from GenBank, and the Bayesian stochastic search variable selection algorithm was employed to analyzed the evolution and origin of circovirus.

**Results:**

Here, the evolutionary origin, mode of transmission, and genetic recombination of the circovirus were determined based on the available circovirus genome sequences. The origin of circoviruses can be traced back to fish circovirus, which might derive from fish genome, and human contributes to transmission of fish circovirus to other species. Furthermore, mosquitos, ticks, bats, and/or rodents might play a role as intermediate hosts in circovirus intra- and inter-species transmission. Two major lineages (A and B) of circoviruses are identified, and frequent recombination events accelerate their variation and spread. The time to the most recent common ancestor of circoviruses can be traced back to around A.D. 600 and has been evolving at a rate of 10^-4^ substitutions site^-1^ year^-1^ for a long time.

**Discussion:**

These comprehensive findings shed light on the evolutionary origin, population dynamics, transmission model, and genetic recombination of the circovirus providing valuable insights for the development of prevention and control strategies against circovirus infections.

## 1 Introduction

Circoviruses, belonging to the family Circoviridae (https://ictv.global/taxonomy), are non-enveloped, icosahedral symmetrical viruses with a circular single-stranded DNA genome of approximately 1.7–2.2 kb in length. They possess the smallest known viral genome and exhibit conserved genome organization. The replicase (Rep) protein, encoded by open reading frame 1 (ORF1), is involved in virus replication, while the major immunogenic capsid (Cap) protein encoded by ORF2 and is associated with host-protection (1).

Since the identification of the first circovirus, various pathological conditions in domestic animals have been linked to circoviral infections. Human circovirus (HCirV) can cause liver damage, evidenced by high HCirV genome copy numbers in the liver and blood with hepatic cytolysis (2). Canine circovirus is associated with diarrhea in dogs and facilitates parvovirus replication (3). Duck circovirus (DuCV) infects ducks of all ages and was initially reported in Mallard ducks, causing feathering disorders, growth retardation, and thinning, which are similar to the clinical symptoms of beak atrophy and dwarfism syndrome (4, 5). Goose circovirus (GCV) induces severe diarrhea, feather disorders, and short clubbed feathers (6, 7). Pigeon circovirus (PiCV) was first identified in a female racing pigeon that exhibited anorexia, lethargy, emaciation, and pectoral muscle atrophy (8, 9). Porcine circovirus type 1 (PCV1) was first described in 1974 and has been detected in both wild and farmed pigs, furthermore, antibodies against PCV1 were detected in human, mice, and cattle (10–12). Porcine circovirus type 2 (PCV2), identified in 1997, emerged as the predominant pathogen responsible for postweaning multisystemic wasting syndrome (PMWS), causing severe immunosuppression, and even death (13, 14). Furthermore, PCV2 has been detected in various other species such as rodents, insects, canines, ruminants, and shellfish, suggesting a wide host range (15–20). In 2015, a novel circovirus, named porcine circovirus type 3 (PCV3), was identified in aborted fetuses of sows. PCV3 is associated with porcine dermatitis and nephropathy syndrome (PDNS) and reproductive disorder symptoms, including lung collapse or failure, lobular pneumonia, multifocal hemorrhage, enlarged lymph nodes, hyperplasia, liver hyperemia and necrosis, kidney swelling or scattered hemorrhagic foci, and spleen enlargement, and it was detected in other species including cattle (21–24). In 2019, another newly identified circovirus, named porcine circovirus type 4 (PCV4) was found in pigs with clinical disease including PDNS in China (25). PCV4-positive pigs also show some other clinical signs, including fever, respiratory signs, and enteric signs; however, its pathogenicity has not been experimentally validated (26). Recent studies demonstrated that circoviruses isolated in birds can be traced back to fish circoviruses, and the ancestor of roundworm viruses is also a circovirus (27, 28).

Circoviruses have been discovered in multiple species and are responsible for substantial economic losses and poses a threat to public health. However, the origin, transmission, and evolutionary pathway (theory of evolution) of circovirus remains poorly understood. To address these gaps, this study aims to investigate the origin, population dynamics, and spreading patterns of circoviruses by analyzing a comprehensive dataset with an extended collection time window. By shedding light on circovirus origin, transmission models, and evolutionary patterns, this study will provide a valuable theoretical basis for the prevention and control of circovirus infections.

## 2 Materials and methods

### 2.1 Dataset preparation

The complete genome sequences of the circovirus genus were collected, including 4,195 strains of mammalian circovirus (bat, bear, chimpanzee, canine, human, civet, elk, mink, pig, rodent, and whale), 777 strains of avian circovirus (bird, duck, goose, swan, and penguin), 5 strains of mosquito circovirus, 8 strains of fish circovirus (babel, whale, and Silurus glanis), and 8 strains of tick circovirus genomes were downloaded from GenBank (**Supplementary Table S1**) (accessed on April 31, 2023) and annotated with collection year, location, and host when available. Multiple sequence alignments were performed using the fast Fourier transform algorithm, and manual checks and corrections were applied, and poorly aligned sequences and those sequences with premature stop codons or frame-shift mutation were excluded from the analysis (29). The substitution model was selected based on the BIC scores using ModelFinder (30). Molecular clock, population dynamics model, and discrete trait substitution model were determined using the Bayes factor (31). The relaxed lognormal molecular clock (32), skyline population model (33), and symmetric migration rate were selected (34).

### 2.2 Recombination detection

For recombination detection, we used SplitsTree software which conducts phi tests for recombination (35) and RDP 4.0 Beta software with eight methods for recombination detection (36). Phi detection was initially conducted, and if the *p*-value was less than 0.05, seven methods (RDP, CHIMAERA, 3SEQ, MAXCHI, GENECONV, BootScan, and SiScan) in the RDP software were used for further detection (37–43). After removing recombinant strains and recombination regions, the final datasets were obtained for further analysis.

### 2.3 Population parameter estimation/phylodynamic analysis

For the final datasets, a tip-dated serial coalescent analysis was performed using the Bayesian approach implemented in the BEAST V.1.10.4 package. This analysis estimated the time to the evolutionary rate, and population dynamics over time (34, 44). The preliminary analysis of the data is found that a uncorrected relaxed clock (lognormal) was the most appropriate molecular clock for next analysis. Distribution of rate heterogeneity model for most species of circovirus was set as GTR+F+I+G4, except PCV1 (HKY+F+G4), PCV2 (GTR+F+G4), and PCV4 (K2P+G4). In order to better evaluate the evolutionary relationship between different species of circovirus, we chose the Yule model to carry out the following calculation. And Bayesian Skyline (Coalescent) model was used to measure the dynamics of the circovirus population. Three independent runs were operated with a chain length of 1 × 10^10^ generations, and sampling them every 100,000 generations. Convergence was assessed using the Tracer software (v1.6; http://tree.bio.ed.ac.uk/software/tracer/) after a burn-in of 10%. Parameters with an effective sample size greater than 200 were accepted. Parameter estimation was summarized as median and 95% HPD. The Bayesian stochastic search variable selection (BSSVS) was employed to identify the most parsimonious description of the spreading process based on the BF test (34). The temporal signal of data set was assessed using the Bayesian evaluation of temporal signal (BETS). BETS compares the fit of the original heterochronous model (M_het_) (with original sampling times) and the corresponding isochronous model (M_iso_) (with a fixed rate). Marginal likelihood estimation (MLE) was performed using generalized stepping-stone sampling. Parameters were estimated using the MCMC approach implemented in BEAST (version 1.10.4). The BEAGLE library (version 3.1.0) was used to perform massive parallelization on computing architectures. A (log) Bayes factor log(P(Y|M_het_)) 2 log(P(Y|M_iso_)) of at least 5 indicates a strong temporal signal in the data set. To evaluate prediction results, a BF cut-off was applied: BF > 10 indicated very strong support, BF > 5 indicated strong support, and BF > 3 indicated positive support for circovirus spread. The final phylogenetic tree was constructed using FigTree (v.1.4.4) (http://tree.bio.ed.ac.uk/software/figtree/) and iTOL (https://itol.embl.de/) software.

### 2.4 Phylogenetic analysis

The complete data set without recombinant sequences was assessed for strength of phylogenetic signal by applying the likelihood mapping approach implemented in IQ-TREE (version 1.6.12) and MEGA 7.0 (81, 82). The nucleotide substitution saturation was estimated using Xia’s test as implemented in the DAMBE (version 7.3.5). Xia’s test compares an observed index of saturation substitution (I_ss_) to a critical index of substitution saturation (I_ss.c_). If Iss is significantly less than Iss.c, data will be considered as little saturation in transition and transversion, and data are good for phylogenetic analysis. The final phylogenetic tree was visualized using FigTree software (v.1.4.4) (https://github.com/rambaut/figtree/releases) and iTOL (https://itol.embl.de).

## 3 Results

### 3.1 Global distribution of animal circovirus

**Figure 1:**
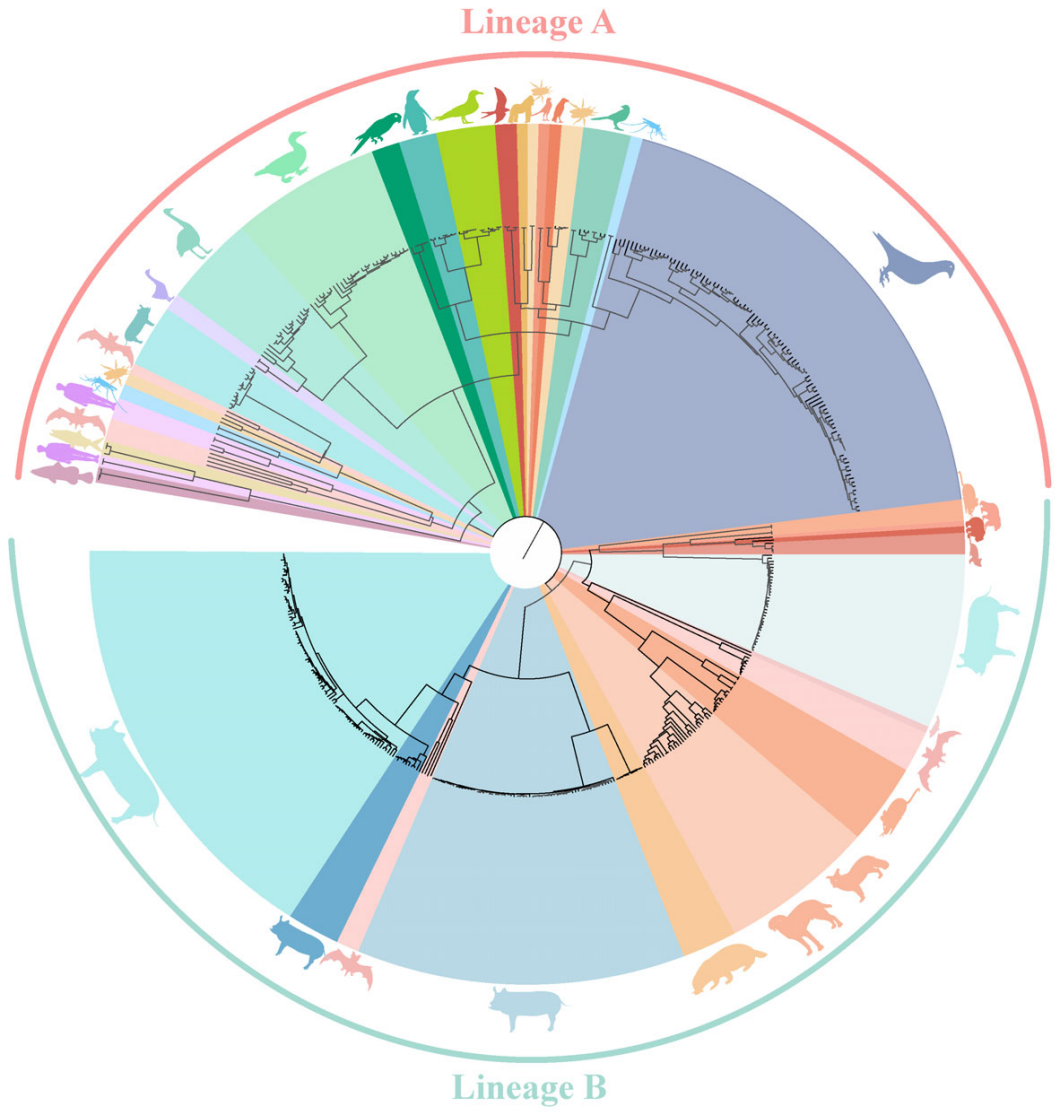
Phylogenetic tree of known circoviruses. The full-length genome sequences of circoviruses publicly available in GenBank was analyzed using BEAST 1.6, with 1,000,000,000 replicates. Different colors represent different circovirus lineages, Lineage A (Red) and B (Green). Different species are also marked with corresponding animal images.

Circoviruses have been discovered in multiple species, with a total of 4,997 strains complete genome sequences from 19 species (mammals, aquatic animal, avians, and insects) analyzed in this study. Using the Bayesian algorithm and Maximum likelihood algorithm, it was observed that circoviruses are classified into two lineages, Lineage A and B (**Figure 1**, **Supplementary Figure S1**, **Supporting Information**). Lineage A includes avian circoviruses (duck-, goose-, swan-, bird-, penguin-, and pigeon-derived animal circoviruses) and circoviruses identified in aquatic animals. Lineage B consists of porcine, canine, fox, mink, mouse, and bat circoviruses, it also contains aquatic mammalian and beaked whale circoviruses. Notably, all currently known HCirV and PCV2 belong to either the Lineage A or B genera, and bat circovirus, as major intermediate hosts, are distributed in both lineages.

**Figure 2:**
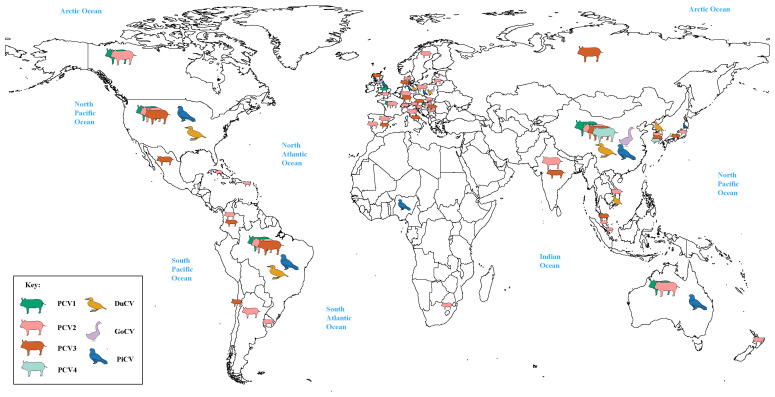
Global distribution of circoviruses. The colors green, pink, brown, light green, yellow, purple, and blue correspond to porcine circovirus 1 (PCV1), porcine circovirus 2 (PCV2), porcine circovirus 3 (PCV3), porcine circovirus 4 (PCV4), duck circovirus (DuCV), goose circovirus (GoCV), and pigeon circovirus (PiCV), respectively.

Meanwhile, it can be clearly observed that DuCV, GCV, PiCV, and PCVs were found to be the most widespread types of circoviruses (**Figure 1**). DuCV has a global distribution, first identified in Germany in 2003 and spreading to other regions in Europe (Poland), Americas (America and Brazil), and Asia (China, South Korea, and Vietnam) (**Figure 2**). GCV, identified in Germany in 1999, has regional distribution in Europe (Poland, Hungary, and the United Kingdom) and Asia (China) (**Figure 2**). PiCV, first recognized in Canada and Australia, has been found on all continents except Antarctica due to the long-distance flight capability of pigeons (**Figure 2**). PCV1, first identified in 1974, has spread in countries with developed pig farming, including the United States, Australia, Brazil, Canada, China, France, Hungary, and the United Kingdom (**Figure 2**). PCV2 was first identified in 1997 in western Canada and has been reported in dozens of countries worldwide (Argentina, Austria, Australia, the United States, Belgium, Brazil, Canada, China, Cuba, Croatia, Colombia, Denmark, France, Germany, Hungary, India, Italy, Japan, Poland, Portugal, Saint Kitts and Nevis, Singapore, Serbia, Spain, Sweden, Slovakia, South Korea, South Africa, Switzerland, Thailand, Malaysia, Netherlands, the United Kingdom, Vietnam, and Uruguay), indicating a global distribution (**Figure 2**). PCV3 was first identified in 2015 in the United States, though it has only been discovered for eight years, positive cases have been found and reported in the Americas (Brazil, Mexico, and, Colombia), Asia (China, South Korea, Japan, Thailand, Russia, Malaysia, and India), and Europe (Italy, Spain, Denmark, Germany, Hungary, Sweden, and Serbia), indicating a global distribution (**Figure 2**). PCV4, first identified in China in 2015, has a current low prevalence in China and South Korea (**Figure 2**).

### 3.2 Genetic recombination in animal circovirus

**Figure 3:**
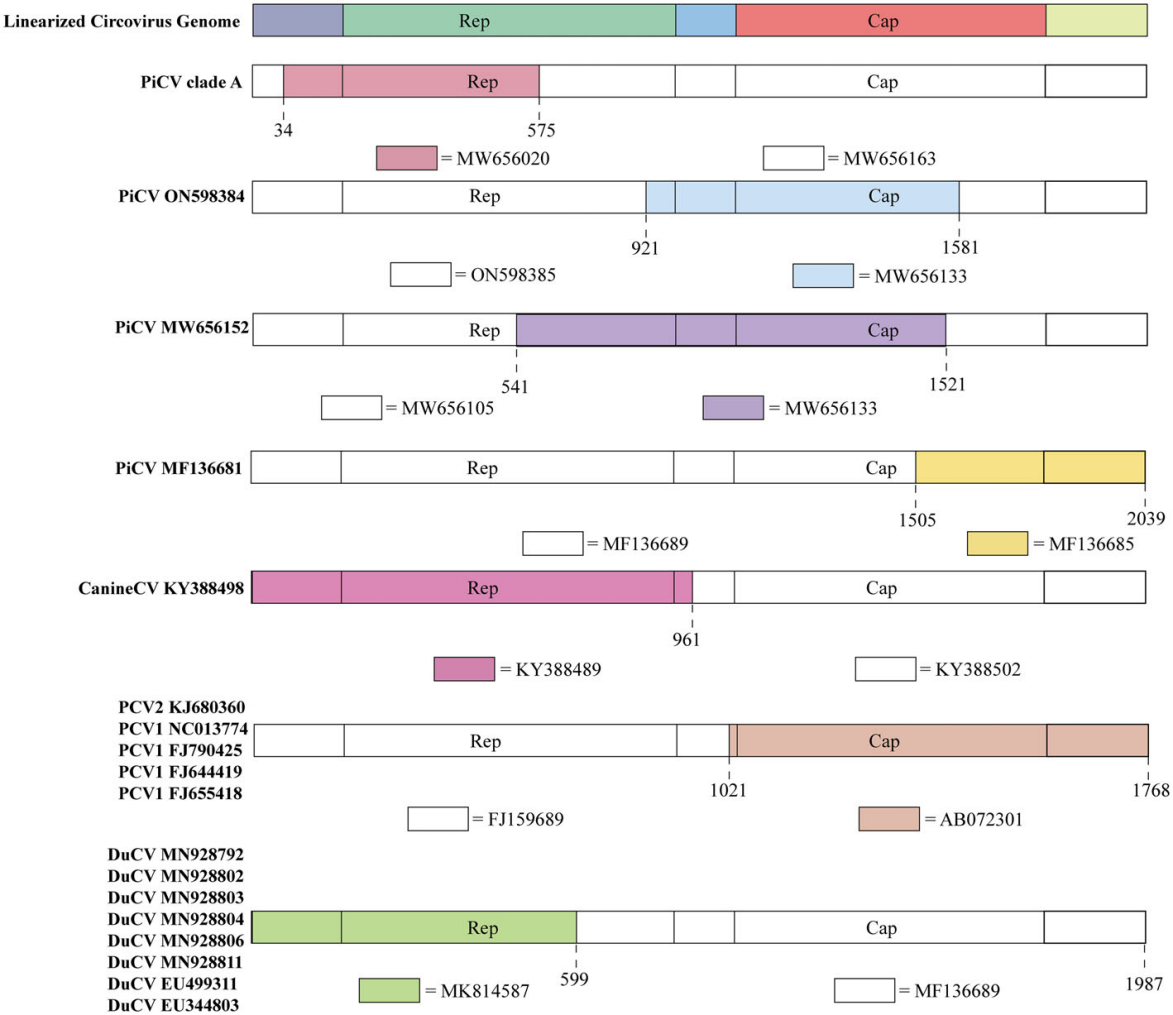
Recombination events in circoviruses. The colored regions in each animal circovirus represents genetic recombination at the genome sites with zoonotic circoviruses. Specific nucleotide locations where recombination occurred are shown as broken lines.

Recombination events have a negative impact on computer algorithms used in traceability research. In order to avoid adverse effects of the recombinant strain on the subsequent analysis, the recombinant strain needs to be excluded from the analysis. We employed RDP4 software to analyze recombination using full genomes. Several circovirus genome sequences exhibited recombination (**Figure 3**).

PiCV diverged into seven distinct clades (A–G) based on the *Cap* gene. Clade A emerged through recombination between MW656163 (Pigeon, Poland, 2020) and MW656020 (Pigeon, Poland, 2020) at a breakpoint of 34–575 bp in the *Rep* gene (**Figure 3**). The recombination region of PiCV was present in both the *Rep* and *Cap* genes, and the PiCV strain MF136681 (Pigeon, Australia, 2023) emerged by recombination between MF136689 (Pigeon, Australia, 2023) and MF136685 (Pigeon, Australia, 2023) at the breakpoint of 1505–2039 (**Figure 3**). Furthermore, we detected recombination in both the *Cap* and *Rep* genes of two PiCV strains (ON598384 (Pigeon, China, 2021) and MW656152 (Pigeon, Poland, 2020)), with breakpoints at 921–1581 and 541–1521, respectively (**Figure 3**). PiCV exhibits the highest recombination frequency among circoviruses, suggesting constant mutations within pigeons, which accelerates the spread of the circovirus and poses a risk of cross-species transmission.

Recombination events were observed in different PCVs. PCV2 strains KJ680360 (Pig, China, 2012) and PCV1 (NC013774 (Pig, Canada, 2008), FJ790425 (Pig, Canada, 2009), FJ644419 (Pig, Canada, 2008), and FJ655418 (Pig, Canada, 2008)) originated through recombination between FJ159689 (Pig, China, 2007) and AB072301 (Pig, Japan, 2001), sharing the same breakpoints of 1021–1768 (**Figure 3**). The current design strategy for PCV2 chimeric vaccines involves replacing the *Cap* gene of PCV1 with the *Cap* gene of PCV2, which is consistent with the recombination region identified in the recombinant strains. Therefore, these recombinant PCV strains may be vaccine viruses, indicating that the recombination events between PCV1 and PCV2 might be caused by human intervention (45).

DuCV also exhibits potential for genetic recombination. An example is the recombination between the MF136689 (Duck, Australia, 2013) and MK814587 (Duck, China, 2019) strains at breakpoints 599–1987. DuCV recombinant strains include MN928792 (Duck, China, 2018), MN928802 (Duck, China, 2018), MN928803 (Duck, China, 2018), MN928804 (Duck, China, 2018), MN928806 (Duck, China, 2018), MN928811 (Duck, China, 2018), EU499311 (Duck, China, 2008), and EU344803 (Duck, China, 2007) (**Figure 3**).

Recombination was also detected in canine circoviruses, where recombination occurred between KY388489 (Dog, China, 2014) and KY388502 (Dog, China, 2015), both from China, with a breakpoint at the *Cap* gene (961–2063) (**Figure 3**).

In addition, some recombination events were also found (MK604479 (Pig, China, 2016), KY437725 (Pig, China, 2015), KP824722 (Pig, China, 2015), KJ679446 (Pig, Germany, 2012), MH465488 (Pig, China, 2018), KX929004 (Pig, China, 2015), and KC415249 (Pig, China, 2008)), but these recombination events were only detected in one parent, lacking the data of another parent. More in-depth analysis needs to be carried out after the increase of virus strain information in the future.

### 3.3 Evolutionary and phylogenetic dynamic of animal circovirus

Bayesian analysis of complete circovirus genomes provided insights into the time of the most recent common ancestor (tMRCA), evolutionary rate, and trends in effective population size.

**Figure 4:**
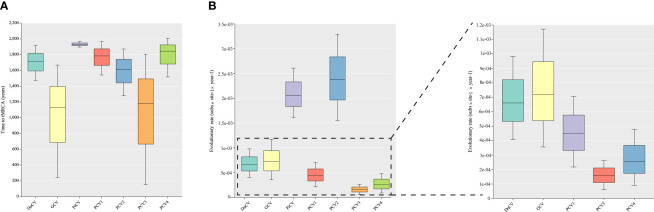
Estimation of the time to the most recent common ancestor (tMRCA) and substitution rates in duck circovirus (DuCV), goose circovirus (GCV), pigeon circovirus (PiCV), porcine circovirus 1 (PCV1), porcine circovirus 2 (PCV2), porcine circovirus 3 (PCV3), and porcine circovirus 4 (PCV4) complete genomes using BEAST (v.1.10.4). **(A)** Time to tMRCA for different species of circoviruses. **(B)** Substitution rates of different species of circoviruses.

**Figure 5:**
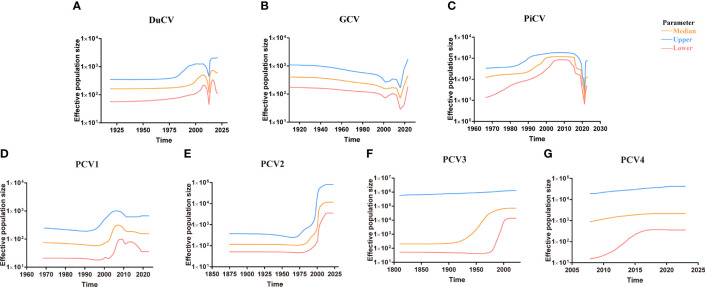
Circovirus population dynamics reconstructed based on complete genome datasets of **(A)** duck circovirus (DuCV), **(B)** goose circovirus (GCV), **(C)** pigeon circovirus (PiCV), **(D)** porcine circovirus 1 (PCV1), **(E)** porcine circovirus 2 (PCV2), **(F)** porcine circovirus 3 (PCV3), and **(G)** porcine circovirus 4 (PCV4). Different colors indicate upper, median, and lower boundaries, representing changes in the effective population size of different circovirus species.

Although DuCV was first reported in 2003, its tMRCA can be traced back to 1715.42, with a nucleotide substitution rate of 6.6 × 10^–4^ substitutions site^–1^ year^–1^ (**Figure 4**). Based on the skyline plot, DuCV experienced population expansion until 2010. Afterward, the effective population size experienced a sharp change, including rapid decrease and increase. After several changes in population dynamics, DuCV’s population size remained the same as it was before 2010 (**Figure 5A**).

The tMRCA and nucleotide substitution rates of the GCV complete genome were 1129.71 and 7.18 × 10^–4^ substitutions site^–1^ year^–1^, respectively (**Figure 4**). Before 2010, it maintained a stable population range, but after 2010, it experienced a rapid decline. The population size began to increase in the past two years and has already exceeded the 2010 level (**Figure 5B**).

PiCV has a fast nucleotide substitution rate of 2.05 × 10^–3^ substitutions site^–1^ year^–1^ and based on the analysis of the tMRCA results (1931.16), it can be found that PiCV is a relatively new circovirus (**Figure 4**). Based on the skyline plot analysis results, it can be observed that PiCV displays similar population trends to the two other types of avian circoviruses (DuCV and GCV). The population size showed a decreased trend between 2010 and 2020; after 2020, the population began to increase (**Figure 5C**). Due to the rapid evolution and high recombination rates of PiCV, it is more susceptible to cross-species transmission, necessitating further in-depth studies.

Until now, PCV1 has spread in countries with developed pig farming, showing a nucleotide rate of 4.46 × 10^–4^ substitutions site^–1^ year^–1^. The tMRCA of PCV1 can be traced back to 1783.291 (**Figures 3**, **4**). Phylogenetic dynamic analysis indicates that the effective population size exhibited a stable growth trend before 2010, then a decreased trend between 2010 and 2020, after 2020 the population size remained relatively small (**Figure 5D**).

The tMRCA of PCV2’s complete genome was 1608.99, with a high nucleotide substitution rate of 2.38 × 10^–3^ substitutions site^–1^ year^–1^ accelerating the emergence of new subgroups, which may cause greater harm (**Figure 4**). Based on the skyline plot results, it can be clearly observed that the population of PCV2 showed rapid expansion before 2018, but plateaued after 2018; the effective population size is maintained a relatively high level (**Figure 5E**).

The PCV3 can be traced back to 1182.203 with a nucleotide substitution rate of 1.59 × 10^-4^ substitutions site^–1^ year^–1^, which proves that PCV3 is a very ancient virus, that predates PCV2 (**Figure 4**). Phylogenetic dynamic analysis indicated that the effective population size of PCV3 expanded rapidly from 1980 to 2018. After 2018, the population expansion plateaued, which is consistent with PCV2, and the effective population size level was similar to PCV2 (**Figure 5F**).

As a newly discovered circular virus, PCV4 is poorly understood. To better understand the phylogenetics of PCV4, ML and NJ methods were used to reconstruct the phylogenies of PCV4 complete genome. Two independent clades were observed in two different tress which displayed similar structures (**Supplementary Figures S2A, B**, **Supporting Information**). The PCV4a clade, included the earliest strain found in China in 2012 (MW600958), could be separated into two individual subclades with stable topological structure, termed PCV4a-1 and PCV4a-2 (**Supplementary Figures S2A, B**, **Supporting Information**). The tMRCA of PCV4 was 1842.916, and the mean substitution rate of the complete genome sequences of all PCV4 strains was 2.56 × 10^−4^ substitutions rate^–1^ year^–1^, which was lower than PCV2 (2.38 × 10^-3^), but close to PCV3 (1.59 × 10^-4^) and PCV1 (4.46 × 10^-4^) (**Figure 4**). The skyline plot result was consistent with the results of PCV2 and 3, it had an expansion trend before 2018 but plateaued after 2018 (**Figure 5G**).

### 3.4 The origin and transmission model of the emergent animal circovirus worldwide

**Figure 6:**
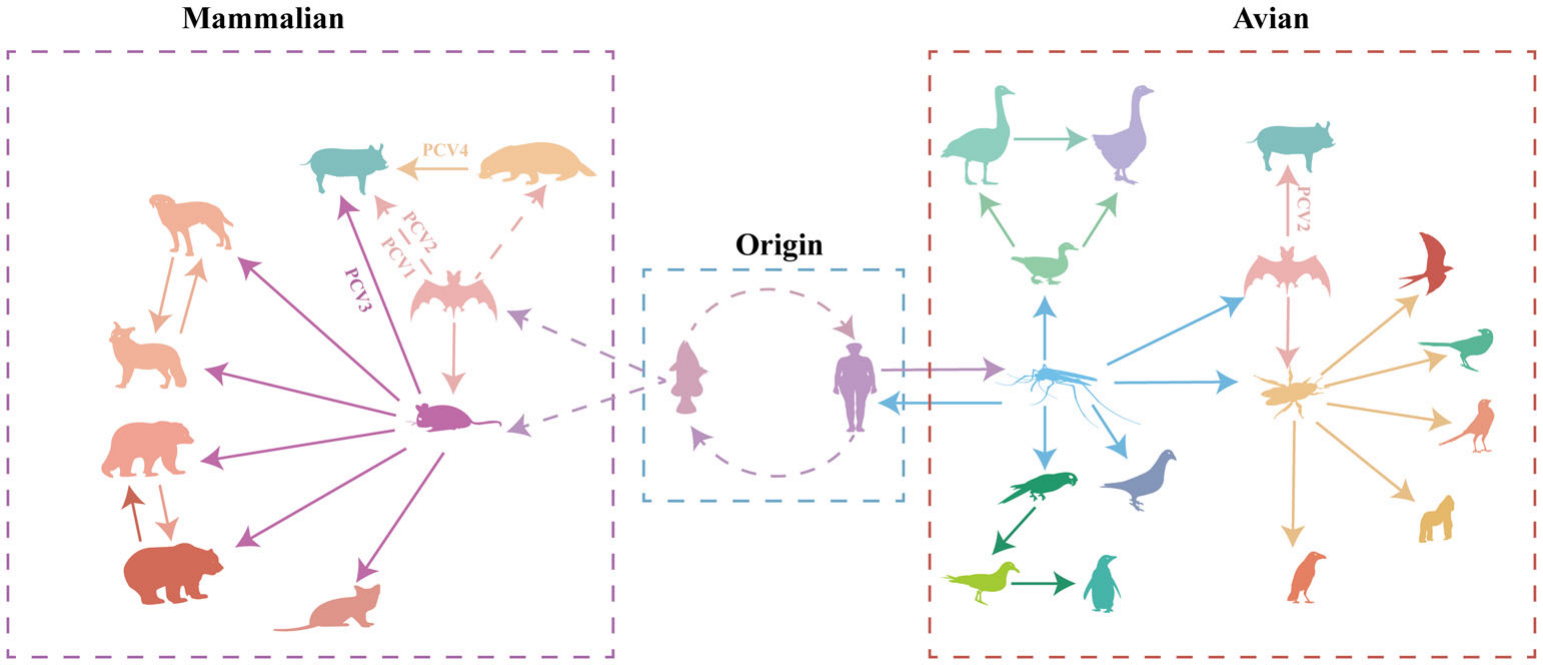
Transmission of circoviruses between different species. Each color represents different species of circovirus. Solid arrows represent direct transmission between the two species in question, while broken arrows represent suspected transmission.

To better understand the origin and transmission process of the circovirus, we analyzed complete circovirus genome sequence information using bioinformatic methods. The origin of circoviruses can be traced back to aquatic animals and humans (**Figure 6**, **Supplementary Figure S3**, **Supporting Information**). Mice and bats, as well as mosquitoes and ticks, serve as intermediate hosts for circovirus transmission in mammals and poultry, respectively (**Figure 6**, **Supplementary Figure S3**, **Supporting Information**). The origin of circoviruses in fish has provoked our interest, and previous research has found that fish plays an important role in endogenous retroviruses (ERV) evolution, promoting the transmission of ERV to amniotic animals (46). To discover potential target viral elements in fish genome, the tBLASTn algorithm was used to screen the potential target elements. (47). *Rep* gene of FCirV was found to possess a 98%-100% similarity to chromosome 1 in fish genome, and *Cap* gene of FCirV also showed high similarity (82%-98%) to chromosome 7 and X in fish genome. During the long-term evolution of aquatic animals, frequent recombination and fragment exchange between genomes led to the emergence of the first type of FCirV, which could be traced back to around A.D. 600 (**Supplementary Figure S3**, **Supporting Information**). Meanwhile, we also used tBLASTn algorithm to compare HCirV and FCirV, and found that they share a *Cap* and *Rep* gene similarity of 97% and 96%, respectively. This was confirmed by phylogenetic analysis as evidenced by fish and humans locating on the same clade (at the tree root) of the multispecies circovirus phylogenetic tree. These results indicate that the origin of circovirus is closely related to the aquatic animal genome, and that there is a close relationship between FCirV and HCirV.

Based on the analysis results, the ancestors of all PCVs are fish circovirus, but the species and numbers of intermediate host of the different PCVs are different (**Figure 6**, **Supplementary Figure S3**, **Supporting Information**). PCV1 and PCV2 are directly transmitted by bats to pig herds (**Figure 6**, **Supplementary Figure S3**, **Supporting Information**). PCV3 was first transmitted by bats to rodents, and then transmitted to pigs (**Figure 6**, **Supplementary Figure S3**, **Supporting Information**). PCV4 was transmitted by bat circovirus to minks, and then to pig herds (**Figure 6**, **Supplementary Figure S3**, **Supporting Information**). PCV1 is currently considered to be non-pathogenic, while PCV2 and PCV3 pose a huge threat to the pig population, the virulence of PCV4 is still unclear; Whether the transmission of circoviruses through different intermediate hosts causes the differences in virulence needs to be verified.

## 4 Discussion

Since the first circovirus was identified in 1974 (10), most studies have focused on the genetic characterization of individual isolates and on clinical and epidemiological investigations. Here, we provide important insights into the origin, phylogenetic dynamic, and evolution process of animal circoviruses.

Previous studies suggest that the circovirus comes from the bat circovirus (5, 48). However, this study presents a more accurate determination of the origin of animal circovirus and proposes an evolutionary pathway (theory of evolution) that explains the emergence of animal circovirus worldwide. The findings indicate that the earliest origin of circovirus can be traced back to fish circovirus, there is a high homology between the FCirV and fish genome. The high similarity between the FCirV genome sequence and the fish genome can be explained by the mechanisms of circovirus replication. Animal genomes contain ssDNA virus-like sequences that resemble the circovirus rolling circle replication initiation protein gene, and eukaryotic genomes also have ssDNA *Rep* gene homologs (49, 50). The Rep protein can recognize DNA sequences in the host genome similar to the circovirus genome’s origin of replication (hairpin loop) and can ligate RNA during the rolling circle replication of the ssDNA virus genome (50, 51). Another hypothesis suggests that the ssDNA virus can capture RNA virus sequence from host genome (52). An mRNA from a coinfecting virus is used as a primer for first or second-strand replication. The recombination of RNA-DNA would have to be followed by reverse transcription, presumably by a cellular reverse transcriptase (53). Recombination of the *Rep* and *Cap* gene is expected to occur. *Cap* gene can incorporate different nucleic acid genomes, and are critical for determining host range and evading host defenses (54, 55). Many ssDNA virus genomes contain unique *Cap* genes, which may originate from uncharacterized RNA viruses (56, 57). In general, positive-stranded ssDNA and ssRNA viruses were much more prone to having chimeric genes than negative-stranded ssRNA virus (51), the frequency of this deep recombination is unknown and is a critical area for future research.

Where there is water, there is life. All life activities originate from water. This shows that humans have a close relationship with water and aquatic animals. This close contact with aquatic animal provides an opportunity for the transmission of viruses across species. Consumption of meat and exposure to animal or human feces increase the chances of circovirus transmission (58). Wetlands are transitional zones between land and water, and as such have an ecosystem with special structures and functions (59). They serve as the cradle of abundant biodiversity in nature and play a vital role in the survival and development of human society. Wetlands are home to a diverse array of organisms, including aquatic animals such as fish, arthropods like mosquitoes, mammals including humans, bats, and mice, various plant species, amphibians, and a wide range of microorganisms. This intricate web of life facilitates significant interactions and contact between different animal species. The spread of circoviruses among poultry is facilitated by Lineage A circoviruses, which include avian and mosquito circoviruses. Mosquitos serve as an important intermediate host for various viruses, and have antiviral mechanisms to limit viral replication (60, 61). In addition, relevant studies have proved that complementing incomplete virus particles can co-infect mosquitos and result in the reconstitution of infectious virus that are able to disseminate into the mosquito salivary glands, suggesting that incomplete particles may play a significant role within hosts and between hosts transmission, reminiscent of the infection cycle of multipartite viruses (62). Mosquitoes play an essential role in the transmission of circoviruses. During the process of circovirus spread, mosquitoes serve as intermediate hosts. Once infection with a circovirus, mosquitoes undergo constant variations due to their antiviral characteristics, ultimately giving rise to a circovirus capable of infecting birds and waterfowl. Through bird and waterfowl migration, circoviruses can rapidly disseminate to different regions and adapt to infect ticks and bats during transmission. Ticks, being highly specialized hematophagous ectoparasites, primarily parasitize avian and mammalian species (63, 64). They also serve as important vectors for transmitting various pathogens, including viruses. Birds also have the potential to transport tick-borne pathogens by carrying infected ticks. This mode of transmission facilitates the circovirus’s rapid spread, surpassing barriers such as fences, mountains, glaciers, deserts, and oceans, which are typically challenging for ticks to traverse (65). Overall, mosquitoes and ticks play important intermediate host roles in avian circovirus transmission.

To understand the factors that accelerate circoviral transmission in mammals, we examine mice and bats as intermediate hosts. In recent decades, several highly impactful zoonotic disease outbreaks, such as Hendra virus, Ebolavirus, Nipah virus, and coronaviruses, have been linked to bat-borne viruses (66–68). As the most abundant, diverse, and geographically dispersed vertebrates, bats have been found to carry 61 viruses (69). Being ancient mammals, bats are hypothesized to possess highly conserved cellular receptors that facilitate the transmission of viruses to other mammals (66). Bats spread viruses to other animals through their feeding habits. Due to the aerodynamics of flight, bats cannot consume large amounts of food. They rely on fruits for energy, but their feeding process involves chewing the fruits to extract sugars and high-energy components, followed by spitting out partially digested fruit remnants that drop to the ground. Subsequently, other animals, including rodents, can ingest these fruit remnants and become infected by viral particles present in the bat saliva, which constitutes the transmission rout of SARS-CoV (70). Rodents carry more pathogens than bats and can act as a reservoir for emerging viruses (69, 71). As mentioned earlier, mice and mink could ingest fruits remnants dropped by bats, leading to their infection with bat circovirus. As a result, mice and minks bring the virus closer to human settlements, potentially leading to the infection of economic or companion animals like pigs and dogs. Thus, bats and rodents serve as reservoirs to expedite the transmission of circoviruses to mammals. These findings demonstrate that various circoviruses from mammals and avians are currently circulating in the wild, providing ample opportunities for circoviruses to undergo evolution and genetic recombination, potentially resulting in recombinant circoviruses that may be more virulent to animals, including humans. As a mammalian circovirus, PCV2 was expected to belong to Lineage B. Notably, our analysis revealed that PCV2 exists in both Lineages. Further investigation into the origin of PCV2 unveiled that both lineages derived from bat circoviruses. The difference lies in the fact that the bat circovirus in Lineage A may have directly originated from human or fish circoviruses, while the bat circovirus in Lineage B was derived from a circovirus carried by mosquitoes. This suggests a close relationship between the origin of PCV2 and bat circovirus, with a less intimate connection to earlier ancestors. While PCV1 shares similarities with PCV2, which directly descends from bat circovirus, PCV3 and PCV4 are not directly generated from pig herds infected by bat circovirus but involve other intermediate hosts. The transmission of PCV3 is closely associated with rodents, whereas PCV4 is linked to minks. These findings indicate that pigs serve as the final hosts, but different intermediate hosts give rise to distinct types of PCVs, which may also contribute to changes in their virulence.

Phylodynamic analysis revealed the earliest origin time, evolution rate, and population dynamics of various circovirus. Circoviruses have been found to be ancient viruses, dating back to approximately A.D. 600, and they undergo mutation rates approximately 10^–4^ substitutions site^–1^ year^–1^ over the long term. The population dynamics of avian circoviruses are influenced by their hosts prior to 2010, the population of avian circovirus remained stable. However, after 2010, there was a rapid decline, likely due to the outbreak of highly pathogenic avian influenza during that period (**Figures 5A–C**) (72–75). Most countries implemented culling measures as a means of prevention and control, which resulted in the loss of viable hosts for avian circoviruses and a subsequent decline in their population. Although highly pathogenic avian influenza remains a global threat, most countries have switched control measures from culling to vaccination, resulting in growing avian populations. This indicates that the host population size of avian circovirus infection is constantly expanding, however, there is currently no vaccine against avian circovirus, which has led to the expansion of the avian circovirus population in recent years (**Figures 5A–C**). This phenomenon also occurred in PCV species. Before 2018, the population of PCV showed an increasing trend, but after 2018, it plateaued (**Figures 5D–G**). This may be related to the outbreak of African swine fever virus (ASFV) in 2018 (76). To prevent the spread of ASFV, countries and regions around the world have implemented measures such as culling and improving biosafety prevention and control. These measures not only curtailed the spread of ASFV but also hindered the transmission of other pig-related viruses.

Recombination occurs during the long-term evolution of viruses. While the recombination frequency of DNA viruses is generally lower than that of RNA viruses, circoviruses have been found to undergo recombination, with avian circovirus displaying a higher recombination compared to mammalian circovirus (**Figure 3**). During viral replication, a set of sub-genomic RNAs is generated, increasing the homologous recombination rate among closely related genes from different lineages of circoviruses or other hosts/viruses through template switching (77). The circulation of viruses among multiple host species contributes to an increased rate of recombination. However, the precise mechanism of genetic recombination in circoviruses remains unclear, including the “breakpoints” in the viral genome where recombination occurs and the specific crossing points of recombinant genes between different viral strains or genotypes. These recombinant strains have different breakpoints (77). In addition, human factors may lead to the emergence of recombinant strains. PCV2 chimeric vaccines, which was based on the genomic backbone of the non-pathogenic PCV1 with the ORF2 *Cap* gene replaced by that of PCV2, have inadvertently led to the detection of chimeric PCV1–2 viruses in the field due to incomplete vaccine inactivation (45, 78). Recombination increases virus genetic diversity and may contribute to the adaptation of a new host (79, 80), and circoviruses have a high frequency of homologous DNA recombination possibly mediated by random template switching during genome replication thought to be mediated by a ‘copy choice’ mechanism of the virus itself.

Overall, this study provides an updated understanding of the origin, population dynamics, and evolution of circoviruses, highlighting the generation of novel viruses with high genetic diversity, and unpredictable changes in virulence and cross-species transmission during avian, aquatic anima, tardigrade, parasite, and mammalian infections. With multiple species of circoviruses circulating in the wild amongst different animal species that may constantly interact with one another, it is a matter of when not if, the next recombinant circovirus will emerge and cause another outbreak in mammals including the human population. These findings contribute to the assessment of circoviruses relevance for swine, avian, and fish industries and may aid in the planning of effective control strategies. It must be emphasized that this work just scratched the surface of circovirus history, biology, future, and constant re-evaluation of the present results will be mandatory to update and improve our understanding of this emergent viral behavior.

## Data availability statement

The datasets presented in this study can be found in online repositories. The names of the repository/repositories and accession number(s) can be found in the article/**Supplementary Material**.

## Author contributions

YC: Conceptualization, Formal Analysis, Investigation, Methodology, Software, Writing – original draft, Writing – review & editing. SL: Conceptualization, Investigation, Writing – original draft. WX: Formal Analysis, Methodology, Writing – original draft. JX: Data curation, Software, Writing – original draft. DW: Data curation, Writing – original draft. LH: Supervision, Writing – original draft. JZ: Formal Analysis, Writing – original draft. XF: Data curation, Writing – original draft. JL: Funding acquisition, Supervision, Writing – review & editing.

## Supplementary material

Supplementary Figure 1 ML Phylogenetic tree of known circoviruses. The full-length genome sequences of circoviruses publicly available in GenBank was analyzed using IQ-TREE software. Different species were marked by different colors.

Supplementary Figure 2 Phylogenetic analysis of the available complete genome of porcine circovirus 4 (PCV4). **(A)** Maximum likelihood tree reconstructed using IQ-Tree. **(B)** Neighbor-joining tree reconstructed using MEGA 6.0. Different clades are represented by different colors as indicated in the figures.

Supplementary Figure 3 Relationships among different species of circoviruses based on complete genome analysis. The MCC tree was reconstructed using BEAST (v.1.10.4), displaying ancestor hosts indicated by posterior probability. Different colored lines represent different species.

Supplementary Table 1 The strain information of the circovirus used in this study.
