# Supplementary material for: Intra- and inter-host origin, evolution dynamics and spatial-temporal transmission characteristics of circoviruses
Source: Front Immunol. 2024 Aug 2;15:1332444. doi: 10.3389/fimmu.2024.1332444 (PMC11327096; doi:10.3389/fimmu.2024.1332444)

Figure S1

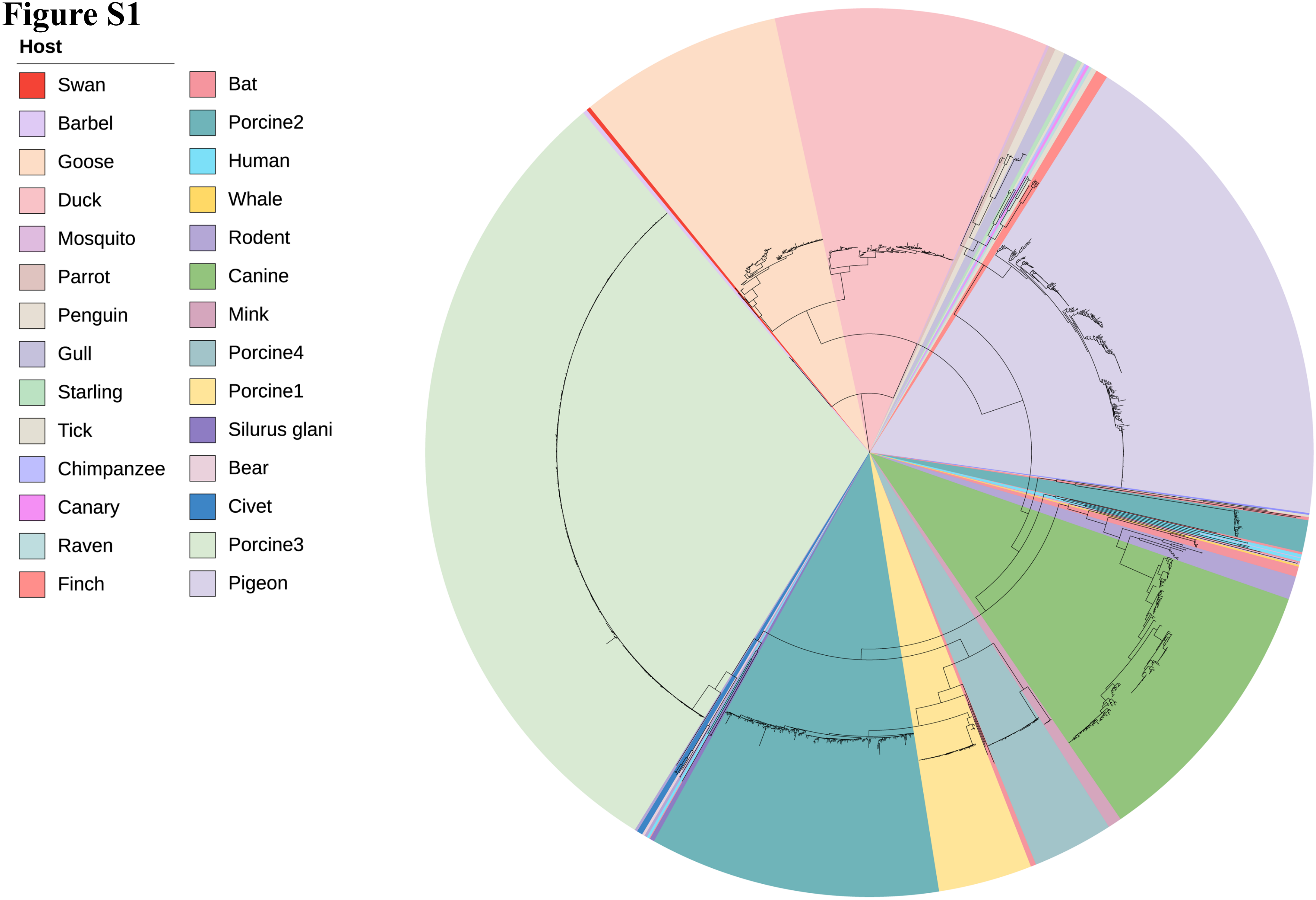
Host
Swan
Barbel
Goose
Duck
Mosquito
Parrot
Penguin
Gull
Starling
Tick
Chimpanzee
Canary
Raven
Finch
Bat
Porcine2
Human
Whale
Rodent
Canine
Mink
Porcine4
Porcine1
Silurus glani
Bear
Civet
Porcine3
Pigeon

Figure S2A

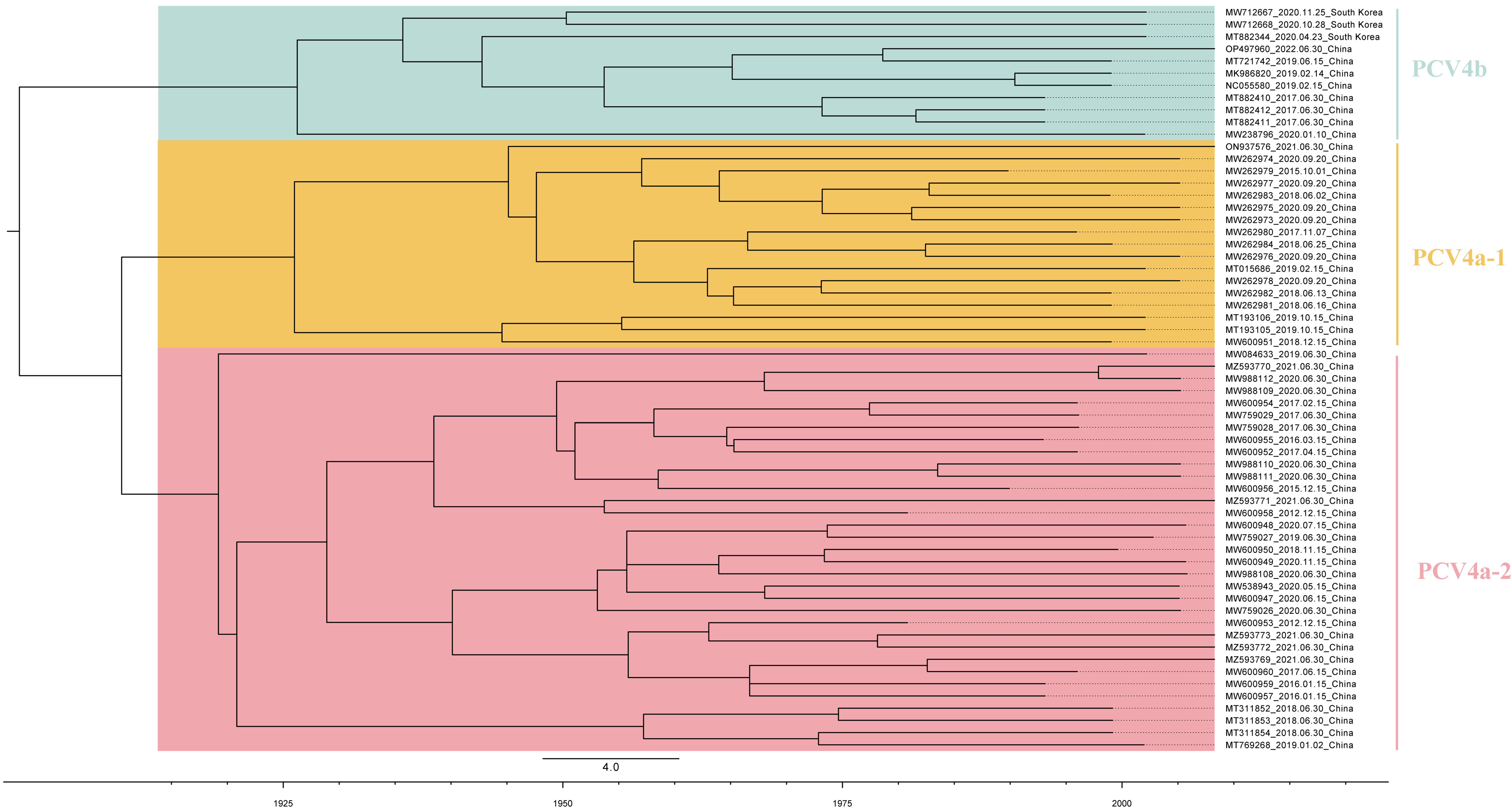
MW712667_2020.11.25_South Korea
MW712668_2020.10.28_South Korea
MT882344_2020.04.23_South Korea
OP497960_2022.06.30_China
MT721742_2019.06.15_China
MK986820_2019.02.14_China
NC055580_2019.02.15_China
MT882410_2017.06.30_China
MT882412_2017.06.30_China
MT882411_2017.06.30_China
MW238796_2020.01.10_China
ON937576_2021.06.30_China
MW262974_2020.09.20_China
MW262979_2015.10.01_China
MW262977_2020.09.20_China
MW262983_2018.06.02_China
MW262975_2020.09.20_China
MW262973_2020.09.20_China
MW262980_2017.11.07_China
MW262984_2018.06.25_China
MW262976_2020.09.20_China
MT015686_2019.02.15_China
MW262978_2020.09.20_China
MW262982_2018.06.13_China
MW262981_2018.06.16_China
MT193106_2019.10.15_China
MT193105_2019.10.15_China
MW600951_2018.12.15_China
MW084633_2019.06.30_China
MZ593770_2021.06.30_China
MW988112_2020.06.30_China
MW988109_2020.06.30_China
MW600954_2017.02.15_China
MW759029_2017.06.30_China
MW759028_2017.06.30_China
MW600955_2016.03.15_China
MW600952_2017.04.15_China
MW988110_2020.06.30_China
MW988111_2020.06.30_China
MW600956_2015.12.15_China
MZ593771_2021.06.30_China
MW600958_2012.12.15_China
MW600948_2020.07.15_China
MW759027_2019.06.30_China
MW600950_2018.11.15_China
MW600949_2020.11.15_China
MW988108_2020.06.30_China
MW538943_2020.05.15_China
MW600947_2020.06.15_China
MW759026_2020.06.30_China
MW600953_2012.12.15_China
MZ593773_2021.06.30_China
MZ593772_2021.06.30_China
MZ593769_2021.06.30_China
MW600960_2017.06.15_China
MW600959_2016.01.15_China
MW600957_2016.01.15_China
MT311852_2018.06.30_China
MT311853_2018.06.30_China
MT311854_2018.06.30_China
MT769268_2019.01.02_China
PCV4b
PCV4a-1
PCV4a-2
4.0
1925
1950
1975
2000

Figure S2B

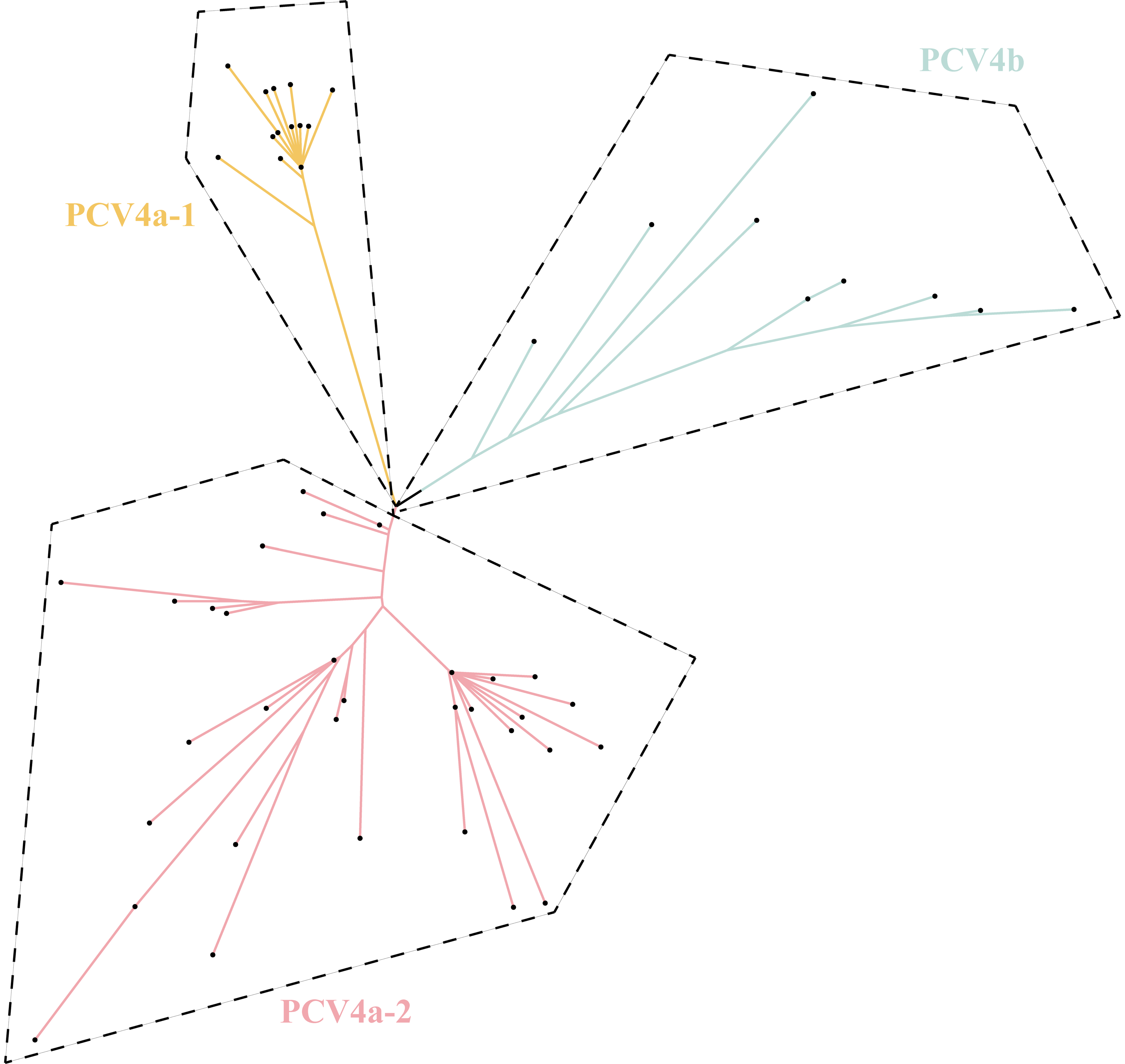

Figure S3

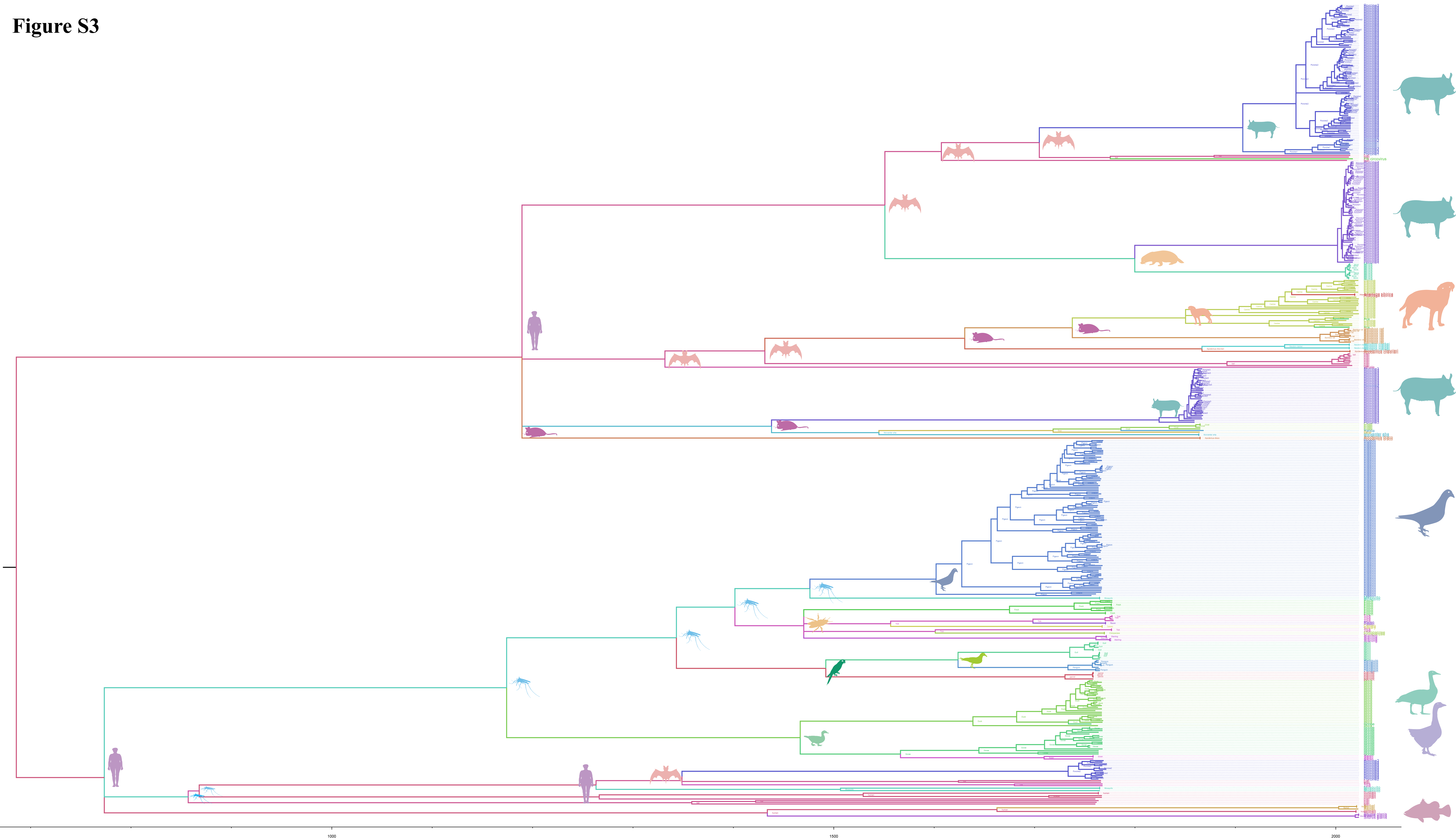

Supplement: Supplementary Figure 1 — ML Phylogenetic tree of known circoviruses. The full-length genome sequences of circoviruses publicly available in GenBank was analyzed using IQ-TREE software. Different species were marked by different colors. [file DataSheet_1.pdf]
